# Exploring social media technologies for novice EFL school teachers to collaborate and communicate: A case in the Czech Republic

**DOI:** 10.3389/fpsyg.2022.1010686

**Published:** 2022-09-27

**Authors:** Jinjin Lu, Feifei Han, Tomáš Janík

**Affiliations:** ^1^Academy of Future Education, Xi’an Jiaotong-Liverpool University, Suzhou, China; ^2^Institute for Learning Sciences and Teacher Education (ILSTE), Australian Catholic University, Brisbane, QLD, Australia; ^3^Vice-Dean for Research and Academic Affairs, Faculty of Education, Masaryk University, Brno, Czechia

**Keywords:** Web 2.0 technology, novice teachers, EFL teaching and learning, multicultural learners, education reform

## Abstract

With an increasing number of international schools, traditional EFL teaching methods may not satisfy students’ needs. This study aims to investigate perceptions of social media technologies (e.g., Web 2.0) and willingness to adopt such technologies to collaborate and communicate in multicultural classrooms among novice EFL schoolteachers in the Czech Republic. The participants were 100 novice EFL schoolteachers in Prague and the South Moravian regions of the Czech Republic. The study used a mixed research method consisting of a survey (stage 1) and a semi-structured interview (stage 2). The survey examined the participants’ appraisal and concerns of using social media technologies to collaborate and to communicate as well as the level of willingness to use social media technologies. A hierarchical cluster analysis using participants’ responses regarding their attitudes and behavioural tendency towards using Web 2.0 social media technologies in language classrooms identified three clusters of teachers. The teachers who were most likely to adopt social Web 2.0 technologies were those who had the highest ratings on both appraisals and concerns regarding the use of social media in language classrooms. The results from the semi-structured interviews were consistent with those from the survey. Together, the results from the two stages demonstrated that most pre-service teachers favoured using Web 2.0 technology for collaboration and communication among colleagues and stakeholders in a broader community, but they displayed contrasting levels of appraisal of and concerns towards using social media technologies. Participants believed that this might be due to their different levels of ICT proficiency, workload, and working environment. The political and practical implications in K-12 education in the Czech context are also discussed.

## Introduction

With an increasing number of migrants, the Czech Republic has seen an influx of multicultural and multilingual families, international students, and skilled workers. Traditionally, schoolteachers used face-to-face methods to teach English language courses. However, the Czech Statistical Office (2018) reports that the total number of migrants increased significantly faster in the Czech Republic than in other countries in Eastern and Central Europe. English proficiency is believed to be a key factor for migrants and their children to be engaged in a multicultural community and may influence their academic studies in the Czech Republic. Moreover, schoolteachers used to communicate with parents *via* written records rather than using digital technologies. That is, parents’ only way to know how their children performed in school is from paper records, and feedback given by parents is based on these records as well. In the long term, the lack of efficient communication is not beneficial for building collaborative partnerships with families (Li et al., 2019). In this case, efficient EFL teaching methods need to be considered and prioritised to meet students’ needs so as to promote the lifelong learning of individuals across different educational and career paths (Ministry of Education Youth and Sports, 2007; Czech Ministry of Education, 2014).

In addition to the inefficient communication methods mentioned above, language teachers’ attitudes, such as their willingness, their self-belief in their digital proficiency, and their attendance of professional development programs may influence their choices of using social media technologies in their classrooms. Their motivation and behavioural tendencies have been discussed *via* the Technology Acceptance Model (TAM), which has been widely used to understand teachers’ belief in terms of using technology (Davis, 1989; Teo et al., 2007). Davis (1989) believed that individuals’ job performance could be enhanced *via* their perceptions of usefulness utilising a particular system and the perceived ease of use as “the degree to which a person believes that using a particular system would be free of effort” (p. 320). This model has been used in language education studies in recent years. For example, Liu et al. (2017) found differences in attitudes towards ICT usage among Chinese EFL teachers based on their previous digital experience or belief transmission regarding student learning. Given that knowledge is distinct from beliefs (Calderhead, 1996) and that attitudes towards use vary by technology type and language competency (King and He, 2006; Jin, 2017; Lai et al., 2017), whether the same relationship between teachers’ attitudes and their social media choices (behavioural tendency) can be found remains to be seen. This study is based on the TAM model, which aims to explore the relationships between novice EFL teachers’ attitudes and behavioural tendency to adopt social Web 2.0 technologies in language classrooms, and is set in the Czech Republic. This study will make significant contributions by providing first-hand data for decision-makers in the development of strategic education policy in terms of language teaching pedagogy and teachers’ professional development in the Czech context. In addition, this pioneering study will serve as a guide for future researchers who undertake projects in a similar historical-cultural context. Specifically, this study is guided by the following research questions:

1. How do Czech novice English teachers’ attitudes towards and behavioural tendencies regarding the use of social Web 2.0 technologies in language classrooms differ by their self-rating of their information technology proficiency?
2. What are Czech novice English teachers’ profiles in terms of their attitudes towards and behavioural tendencies regarding the use of social Web 2.0 technologies in language classrooms?
3. How are Czech novice English teachers’ self-rating of their information technology proficiency associated with their profiles in terms of their attitudes towards and behavioural tendencies regarding the use of social Web 2.0 technologies in language classrooms?
4. What are the qualitative descriptions of Czech novice English teachers’ attitudes towards the use of social Web 2.0 technologies in language classrooms?

## Literature review

### Teachers’ attitudes towards and behavioural tendencies regarding the use of social media technologies

With the development of digital technology, social media has played a key role in EFL learning and teaching in the 21st century. Teachers’ appraisals of the adoption of social media technologies may increase students’ and teachers’ engagement, collaboration, and communication. For example, Yost and Fan (2014) investigated perceptions of early childhood (EC) educators and teachers regarding the use of Web 2.0 technologies in Australia. The findings showed that most EC educators and teachers held positive attitudes towards the use of Web 2.0 technologies in EC centres because they were able to be more effectively involved in community communication. Jung and Suzuki (2015) used a Wiki in a language programme to encourage and support collaborative constructivist learning. Their research revealed that most participants were satisfied with the adoption of a Wiki in their language learning and found that wiki-based multicultural Japanese language learning is different from the traditional Japanese teaching method. Similarly, Balakrishnan et al. (2015) claimed that university students are receptive to using social media-enabled tools as part of their learning process due to substantial improvements in the self, social influence, and functionality after using social media tools. In Europe, research studies highlight the importance of the usage of digital tools (Crystal, 2000) and support the role of social media technology to revitalise endangered languages (Ferré-Pavia et al., 2018).

However, language teachers’ concerns regarding the use of social media technologies in classrooms have also been identified in previous studies. Researchers found that teachers’ negative perceptions of the adoption of Web 2.0 technologies are correlated with many factors, such as individuals’ digital experience, cultural backgrounds, school context, digital proficiency, and professional training (Penner and Grodek, 2014; Yost and Fan, 2014; Alrasheedi and Capretz, 2015; Jung and Suzuki, 2015; Liu et al., 2017; Gan et al., 2021; Lu, 2022). Studies have indicated that language teachers’ limited adoption of social media technologies in classrooms is attributable to their lack of knowledge regarding how to effectively integrate technology in their teaching practise (Alrasheedi and Capretz, 2015; Gan et al., 2021). Yost and Fan (2014) noted that EC teachers who were more confident and experienced in using social media tools were more likely to use them in EC centres. Jung and Suzuki (2015, p. 836) found that ‘wiki-based learning can be in conflict with the more traditional, didactic ways of teaching and learning’ in Korean schools. Similarly, in China, EFL teachers’ intention to adopt Web 2.0 technologies has been found to be the most important predictive factor in the adoption of social media tools in language classrooms (Mei et al., 2018). A more recent study argues that teachers’ lack of confidence and low level of digital literacy as well as inadequate professional development programs are said to lead to a mismatch between the conception of digital technology and the pedagogy (Li et al., 2019). Li et al. (2019) found that there was a discrepancy between the requirement of the technology skills that language teachers should have and their actual usage in classrooms. Finally, Selwyn et al. (2018) noted that many schools in less developed countries still remain “old-fashioned and pre-digital” -(p. 151) and that many teachers are “principled pragmatists” (p. 152) who have not fully and readily understood the conception of digital technology. In this regard, English language teachers’ experience, digital knowledge, and workplace contexts are pivotal, as these factors may influence their understanding, attitudes, and real adoption of Web 2.0 technology.

### EFL teachers’ professional development programs for the use of social media technologies

Teachers’ professional development has been widely studied due to its impact on the quality of teaching, and it is closely related to students’ learning and achievement (Darling-Hammond, 2012). For EFL teachers, professional development is traditionally focused on formally provided courses, peer observation, seminars, and master programs (Abednia, 2012; Gleeson and Tait, 2012; Xu, 2015). However, “effective professional learning is increasingly to involve teachers sharing knowledge and experience with others” (Lantz-Andersson et al., 2018, p. 303), particularly in the form of “participation in a network of teachers” (OECD, 2014, p. 168). Such networks are regarded as gathered groups of teachers who have a ready source of “knowledge that is situated in the day-to day lived experiences of teachers and best understood through critical reflection with others who share the same experience” (Vescio et al., 2008, p. 81). Conventional sharing experiences and knowledge have been critically argued by Schlager and Fusco (2003) in their review, which states that conventional professional development organised at the school, local, and national levels is “disconnected from practise, fragmented and misaligned” (p. 205). Compared with traditional face-to-face communication and interaction in teachers’ professional development programs, EFL teachers could become more mobile, less constrained by time, and more engaged in the online community.

Although the online community has brought significant benefits to EFL teachers’ professional development, concerns are also discussed in research studies. For example, data security is becoming an alarming factor that might become a barrier to the active involvement of teachers in the online community for the purposes of information sharing, identity construction, and peer interaction (Lai et al., 2011; Yost and Fan, 2014). Furthermore, the dominant role of “structured conversations” moderated by more experienced teachers is also regarded as an obstacle because their dominant positions may lead to less visibility of inexperienced teachers in the online community (Lantz-Andersson et al., 2018). Time-related issues have also been raised as concerns (Marklund, 2015; Rosenberg et al., 2016). Teachers’ busy schedules and heavy workload after regular hours may leave these teachers feeling ‘overwhelmed’ by the regular flow of information (Davis, 2015).

### Using social media technology in EFL teaching in the Czech context

For historical-political reasons, English education programs have not been developed as efficiently as those for Russian and German in the Czech Republic (Nekvapil and Nekula, 2006). Klimova (2014) noted that the main reasons of the lack of the development of efficient EFL teaching strategies were that (1) teachers did not use English fully as the target language in EFL classes (Chodera, 2013), (2) teachers are dominant and learners are more passive in EFL classes (Šebestová et al., 2011), (3) teachers do not pay attention to comprehensive language skill development, and there is a small number of qualified EFL teachers in K–12 contexts (Šebestová et al., 2011; Hrozková, 2013), and (4) EFL teaching methods in the Czech Republic are primarily administered in a traditional face-to-face way with the focus on high-stake examinations (Klimova, 2014). For these reasons, it is essential to explore an efficient way to assist teachers in changing their dominant roles, and improving their communication with students with the support of various English resources is urgent and necessary in the current Czech K–12 context^1^ (Czech Ministry of Education, 2014).

With the expansion of multilingual and multicultural schools in the Czech Republic, innovative EFL teaching methods have aroused the interests of scholars; in particular, using digital technologies in EFL teaching and learning has gained greater attention in this country. In the limited number of English publications related to this field of research in the Czech Republic, the discussion focuses primarily on three aspects: (1) factors that impact teachers using digital technologies (Hrtoňová et al., 2015), (2) the adoption of digital technology at different levels of education (Šumak et al., 2011), and (3) using digital technology in the development of teachers’ professional identity and beliefs (Kutálková, 2017). Compared with the use of digital technology in higher education, its adoption in primary and secondary education depends on teachers’ information literacy, their motivation and initiative, and the quality of equipment provided (Šumak et al., 2011). Beran et al. (2007) noted that teachers held a more conservative attitude towards using digital technology in the Czech Republic than teachers in the broader community. In addition, the motivation regarding and acceptance rate of the use of Web 2.0 technology in lower secondary schools are “relatively low,” as teachers in the Czech Republic believe that it will bring extra work outside of their regular working hours (Kutálková, 2017, p. 1356). This was supported by the study Herout (2017), which found that novice EFL teachers did not use any social networking tools in teaching and learning in schools.

However, a large number of Czech students use social media tools, such as Facebook, Instagram, and online learning platforms, in their daily life, and these social media tools have been gaining increasing popularity among the younger generation (Herout, 2017). Due to the high popularity of the use of social media tools, they should be used for the purpose of formal education and implemented in the curriculum, as this is essential for primary and secondary education in the Czech Republic (Herout, 2016). This gap and mismatch between students and teachers in the use of social media tools in classrooms are not beneficial for communication and interaction in or outside schools. For example, study of Selwyn (2009) highlighted that social media tools, such as Facebook, mobile instant messaging, and other social networking sites, are viewed as affordable open spaces because students are more open to expressing their identity and feel more freedom in communication with a broader audience, including teachers, peers, and community staff.

To summarise, although concerns regarding the use of social media tools have been raised in the above-mentioned literature, we cannot deny that the benefits of using Web 2.0 technologies are significant for enhancing communication with and interaction between students and EFL teachers, improving EFL teachers’ online professional development in an informal way, and being favoured by the younger generation in their daily learning process. However, little research has focused on exploring novice English teachers’ attitudes towards and behavioural tendencies regarding the adoption of Web 2.0 technologies in schools, particularly in the Czech context. In this regard, this study will not only make a significant contribution to fill the gap in the literature but also provide evidence for decision-makers in EFL pedagogy and curriculum development in the Czech context.

## Research methods

A sequential mixed research method was used in this study. Teddlie and Tashakkori (2010) argued that the benefits of using a mixed research method include “the broad inquiry logic that guides the selection of specific methods and that is informed by conceptual positions common to mixed methods practitioners (e.g., the rejection of “either-or” choices at all processes)” (p. 5). The use of mixed research methods yields much richer data than the use of a single research paradigm and allows researchers to obtain more comprehensive insight into the research findings (Johnson and Turner, 2002). Thus, qualitative and quantitative methods can draw on the strengths and minimise the weaknesses both in single studies and across studies rather than being regarded as two extreme poles in the research paradigm.

### Instruments

A questionnaire was adapted based on the previous study undertaken in a multicultural context (Authors, 2015). To ensure content validity, three novice English teachers and three research experts in the field were invited to review all of the questionnaire items to ensure readability, comprehensiveness, and clarity. The two groups of reviewers first had several rounds of discussions on which items should be included and excluded until they finally reached a consensus on all question items for the final questionnaire.

The questionnaire was composed of three sections: (1) background information, (2) participants’ awareness and understandings of the use of social media tools in multicultural classrooms, and (3) open-ended questions. The first part of the questionnaire was designed to obtain participants’ background information. In the second part, a five-point Likert scale ranging from *Strongly Agree* to *Strongly Disagree* was used to investigate participants’ awareness and understanding of using Web 2.0 technologies in language classrooms. The third part was composed of two open-ended questions, which were designed to gain participants’ perceptions and suggestions in terms of further development of the interactive project website.

Considering the use of the questionnaire to obtain quantitative data, a semi-structured interview was adopted that allowed participants more freedom to express their opinions of their understanding of and experiences and concerns with using social media web tools in EFL teaching. Additionally, this form of data collection benefits the researcher by collecting more in-depth data from the participants’ responses through allowing them to elaborate their meaning explicitly (Krueger and Casey, 2000).

### Research procedures

**Figure 1:**
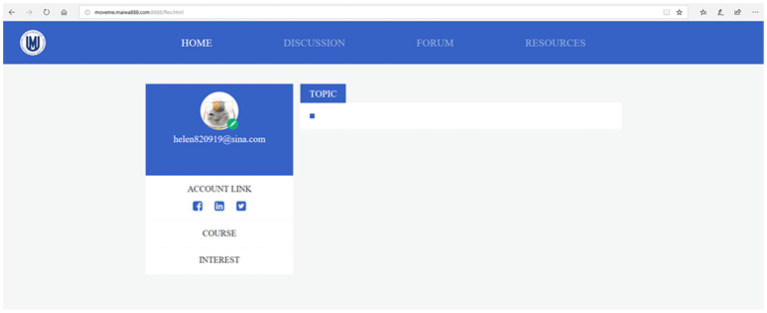
The project website interface.

The research was divided into quantitative and qualitative stages. Through the theoretical lenses, an interactive website was constructed as a means to explore approaches to enabling participants to discover and have access to useful technology, be engaged in multicultural communities, be able to create English teaching and learning contents and express their identities, and interact with a broader social community. In the first stage, K–12 novice English teachers were recruited and invited to attend workshops based on their time schedules in two major multicultural and multilingual cities: Prague and Brno (South Moravian Region). The purpose of the workshops is to introduce the theoretical concepts of the study and explain the benefits of participants’ involvement in the project website (Figure 1). During the workshops, the principal researcher showed the different functions of navigation bars and discussed how to link personal social media tools to interact efficiently with participants engaging with the website. The participants were allowed to explore, interact, and communicate on the project website for two semesters (Fall 2018 and Spring 2019). After the workshops, if the participants wished to join the project, they were required to sign consent forms. All of them fully understood their rights and the process of the project. They were able to interact with peers voluntarily in the project website, and if they were willing, they could share their contact information so that research members could provide support if they had any technical questions in the process. An online survey was sent to all of the participants at the end of the spring semester *via* emails that were provided in the workshops.

During the second stage, a semi-structured interview schedule was developed based on the preferred contact methods given by the participants during the first stage. This form of interview was chosen because it provided “in-depth information pertaining to participants” experiences and viewpoints of a specific topic (Turner, 2010, p. 754). In this study, the 10 focus questions were designed for novice teachers to seek answers to achieve research goals. All of the interview questions were reviewed by local and international experts in the field to ensure that the participants would be able to understand the questions.

### Data analysis

#### Quantitative analyses

In terms of the quantitative analyses, the first step was to explore the factor structure of the questionnaire, which was achieved through performing an exploratory factor analysis (EFA) using the principal component procedure followed varimax rotation, as it was uncertain whether the factors of attitudes and behavioural tendencies are related. The items which had high coefficients loaded across scales were deleted (Field, 2013). To evaluate the internal consistency of each scale, the Cronbach’s alpha reliability was calculated. To answer the first research question—the differences between Czech teachers’ attitudes and behavioural tendencies regarding the use of social Web 2.0 technologies in language classrooms according to their self-ratings of their IT proficiency—a one-way ANOVA was conducted. To answer the second research question—the profiles of Czech teachers’ attitudes towards and behavioural tendencies regarding the use of social Web 2.0 technologies in language classrooms—a hierarchical cluster analysis was performed using the mean scores of the attitudes and behavioural tendency scores, followed by a series of one-way ANOVAs. For the final research question, a cross-tabulation was performed between teachers’ self-ratings of their IT proficiency and their use of the clusters resulting from the hierarchical cluster analysis. All of the analyses were conducted in SPSS version 28.

#### Qualitative analyses

In the qualitative stage, a three-step coding process was adopted to synthesise the common items of the meaning-making process for the textual data (Creswell, 2015). The three-step coding process is based on coding approach of Strauss and Corbin (1998), which allows the researcher to read the raw data first word by word and then line by line to categorise them into various codes. Afterwards, the different codes identified were categorised into themes grounded in the data. The final step was to integrate these themes into categories to form a systematic scheme (Ryan and Bernard, 2000; Grbich, 2007). These rigorous steps ensured that the analysis and output of the data were conducted in a flexible and valid way.

### Participants

After obtaining ethical approval from the university, a post on the project was sent to the K–12 schools in the two regions. All of the participants were contacted by email and signed consent forms to participate in the research project voluntarily. They fully understood their rights and the information regarding the research.

There were 112 participants invited to complete the survey online in the final study, and 100 complete questionnaires were ultimately received. The participants’ information is shown in Table 1.

**Table 1:** Participants’ backgrounds.

	***N***	
	*n*	Percentage (%)
**Gender**		
Male	32	32
Female	68	68
**Length of English teaching experience (year)**		
Less than 1 year	8	8
1–3 years	15	15
3–5 years (not including five)	77	77
**School type**		
International kindergarten	4	4
Public primary and secondary schools	34	34
International primary schools	2	2
Public middle-high schools	32	32
International middle schools	1	1
Other	4	4
**Self-rated knowledge of information technology (IT)**		
Below average	56	2
Poor	8	8
Average	46	46
Above average	44	31
Excellent	13	13

Table 1 shows that most participants have been teaching English for between 3 and 5 years (*n* = 77) in public schools (*n* = 66); however, they self-rated their IT proficiency at only an average level (*n* = 46). After the preliminary collection of questionnaire data, seven participants (P1–P7) were invited *via* email to participate in face-to-face interviews. The interviewees were selected as a homogeneous group, and we believe that the sample size was adequate for qualitative research (Creswell, 1998; Saunders, 2012; Creswell and Poth, 2017). The participants who attended the interviews ranged from 25 to 30 years old and had less than 3 years of teaching experience. Face-to-face interviews lasting approximately 30–40 min were conducted in English by the principal researcher with the help of research assistants using a small audio recorder device. The research assistants were doctoral students in the research group and had previous research project experience. All interviews were conducted in English, as the language teachers were highly confident that they could communicate in English. The audio files were then uploaded onto a password-protected computer and transcribed by the researcher. The transcriptions were distributed to the research team members for quality checking. *Via* several rounds of member checking, the research team ensured that there were no grammatical errors, after which the transcripts were ready for data entry and analysis. The interviewees’ information is listed in Table 2.

**Table 2:** Interviewees’ information.

Participants	Schools	Teaching experience
P1 (Female)	Public primary	1 year
P2 (Female)	Public secondary	6 months
P3 (Female)	Public secondary	8 months
P4 (Male)	International high school	1 year
P5 (Female)	Public primary	2.5 years
P6 (Female)	Public primary	2 years
P7 (Male)	International high school	3 years

## Results

### Results of EFA and the reliability of the scales

The results of EFA (KMO = 0.91) of the questionnaire are presented in Table 3, which show that 13 items were retained, representing three factors: two factors related to teachers’ attitudes, namely appraisal of using social Web 2.0 technologies (three items) and concern of using social Web 2.0 technologies (three items); and one factor describing teachers’ behavioural tendencies regarding the use of social Web 2.0 technologies (seven items). Altogether, these three factors accounted for 71.07% of the total variance. The values of the Cronbach’s alphas showed that the reliability of all three scales was above the acceptable level. The details are shown in Table 3.

**Table 3:** Results of the EFA and reliability of the questionnaire.

Scales	Description of items	Rotated factor loadings		
Appraisal of using social Web 2.0 technologies (α = 0.89)	An interactive website (digital habitat) like this can help enhance the collaboration in teaching practise among English teachers.	0.72		
	An interactive website (digital habitat) like this can help improve my knowledge of technological tools used in English language teaching.	0.54		
	An interactive website (digital habitat) like this can help expand my networks within the English groups domestically and internationally.	0.84		
Concerns regarding the use of social Web 2.0 technologies (α = 0.86)	I am concerned that adoption of social media tools on the interactive website (digital habitat) can be risky.		0.78	
	I am concerned regarding the confidentiality of the information on the interactive website (digital habitat).		0.81	
	I am concerned about the quality of the resources that are provided on the interactive website (digital habitat).		0.76	
Behavioural tendencies regarding the use of social Web 2.0 technologies (α = 0.73)	I am willing to ask questions related to English language teaching and learning on this interactive website (digital habitat).			0.76
	I am willing to answer questions other people propose about English language teaching and learning on this interactive website (digital habitat).			0.75
	I am willing to share resources with others on this interactive website (digital habitat).			0.82
	I am willing to communicate with (other) experienced English teachers using an interactive website like this (digital habitat).			0.77
	I am willing to communicate with (other) parents using an interactive website like this (digital habitat).			0.83
	I am willing to collaborate with (other) school professionals using an interactive website like this (digital habitat).			0.80
	I am willing to collaborate with (other) novice English teachers on an interactive website like this (digital habitat).			0.85

Values less than.50 were removed; KMO: 0.91.

### Results for research question 1: Differences in teachers’ attitudes and behavioural tendencies regarding the use of social Web 2.0 technologies

The results of one-way ANOVAs are presented in Table 4 and show that teachers with low IT proficiency and those with high IT proficiency according to self-rating differed significantly on all the three scales: appraisal of using social Web 2.0 technologies: *F* (1,98) = 12.72, *p* < 0.01, η^2^ = 0.12; concern regarding the use of social Web 2.0 technologies: *F* (1,98) = 13.16, *p* < 0.01, η^2^ = 0.21; and behavioural tendencies regarding the use of social Web 2.0 technologies: *F* (1,98) = 29.72, *p* < 0.01, η^2^ = 0.17. Specifically, teachers who self-rated themselves as having higher IT proficiency also had higher ratings for appraisal and concern regarding the use of social Web 2.0 technologies; moreover, they reported being more likely to adopt social Web 2.0 technologies than teachers who self-rated themselves as having lower IT proficiency.

**Table 4:** Results of one-way ANOVAs by teachers’ self-ratings of their IT proficiency.

Scales	Low IT proficiency (*n* = 56)	High IT proficiency (*n* = 44)	*F*	*p*	η^2^
	*M*	*M*			
Appraisal of using social Web 2.0 technologies	3.58	4.08	12.72	0.00	0.12
Concern regarding the use of social Web 2.0 technologies	3.35	3.90	13.16	0.00	0.12
Behavioural tendencies regarding the use of social Web 2.0 technologies	3.45	3.88	6.52	0.01	0.06

### Results for research question 2: Czech teachers’ profiles in terms of their attitudes and behavioural tendencies regarding the use of social Web 2.0 technologies

Based on the increasing value of the squared Euclidean distance between clusters, a three-cluster solution was produced, with cluster 1 having 54 teachers, cluster 2 having 28 teachers, and cluster 3 having 18 teachers. On the basis of the cluster membership, a series of ANOVAs showed that the three clusters of teachers differed significantly on all three scales: appraisal of using social Web 2.0 technologies: *F* (1,97) = 63.86, *p* < 0.01, η^2^ = 0.12; concern regarding the use of social Web 2.0 technologies: *F* (1,97) = 32.03, *p* < 0.01, η^2^ = 0.12; and behavioural tendencies regarding the use of social Web 2.0 technologies: *F* (1,97) = 94.42, *p* < 0.01, η^2^ = 0.06. The *post-hoc* analyses demonstrate that teachers in cluster 3 were more likely to adopt social Web 2.0 technologies than teachers in clusters 1 and 2. At the same time, they also had the highest ratings for both appraisal of using social Web 2.0 technologies and concern regarding the use of social Web 2.0 technologies compared to those in clusters 1 and 2. Teachers in cluster 1 were more likely to adopt social Web 2.0 technologies than teachers cluster 2. They also had higher ratings for appraisal of using social Web 2.0 technologies than teachers in cluster 2, but they did not differ from teachers in cluster 2 on concern regarding the use of social Web 2.0 technologies. The results of one-way ANOVAs and *post hoc* analyses are presented in Table 5.

**Table 5:** Results of one-way ANOVAs and *post-hoc* analyses based on Czech teachers’ profiles.

Scales	Clusters	*M*	*F*	*p*	η^2^	*Post-hoc*
Appraisal of using social Web 2.0 technologies	cluster 1	3.93	63.86	0.00	0.12	1 > 2
	cluster 2	3.01				1 < 3
	cluster 3	4.65				2 < 3
Concern regarding the use of social Web 2.0 technologies	cluster 1	3.46	32.03	0.00	0.12	1 = 2
	cluster 2	3.18				1 < 3
	cluster 3	4.63				2 < 3
Behavioural tendencies regarding the use of social Web 2.0 technologies	cluster 1	3.83	94.42	0.00	0.06	1 > 2
	cluster 2	2.65				1 < 3
	cluster 3	4.63				2 < 3

### Results for research question 3: The association between Czech teachers’ self-rating of their information technology proficiency and their profiles in terms of their attitudes towards and their behavioural tendency regarding the use of social Web 2.0 technologies in language classrooms

The results of cross-tabulation are presented in Table 6 and show a significant and moderate association between teachers’ self-ratings of their information technology proficiency and their profiles: χ^2^ (2) = 18.49, *p < 0.*01, Cramer’s V = 0.43. Of the three clusters of teachers, the proportions of self-ratings of low and high information technology proficiency did not differ among teachers in clusters 1 and 2. However, among cluster 3 teachers, who reported having the highest tendency to adopt social media Web 2.0 tools, a significantly higher proportion self-rated themselves as having high information technology proficiency (88.9%) than as having low information technology proficiency (11.1%).

**Table 6:** Results of the cross-tabulation analysis.

Teachers’ profiles	Count % within the cluster	Low IT proficiency	High IT proficiency	Total
Cluster 1	Count	34^a^	20^a^	54
	% within the cluster	63.0%	37.0%	100.0%
Cluster 2	Count	20^a^	8^a^	28
	% within the cluster	71.4%	28.6%	100.0%
Cluster 3	Count	2^a^	16^b^	18
	% within the cluster	11.1%	88.9%	100.0%
Total	Count	56	44	100
	% within the cluster	56.0%	44.0%	100.0%

The different subscript letters denote the proportions of the self-rated categories within each cluster that significantly differed from each other at the 0.05 level.

### Results for research question 4: Qualitative descriptions of Czech teachers’ attitudes towards using social Web 2.0 technologies in language classrooms

The qualitative data were obtained from both open-ended questions in the questionnaire and semi-structured interviews. Following the three-step coding process, three themes emerged from the participants’ responses.

### Appraisal of adoption of social media technologies

Within this category, the largest number of responses reflected “Enhancing peer communication” (*n* = 58), followed by “Strengthening novice English teachers” social networks (*n* = 47) and “Obtaining up-to-date information from a broader space” (*n* = 33). The interviewees reported that the rising popularity of smartphones and Facebook, which were streamlined with the project website, might increase their likelihood of adopting such technologies in seeking information and interaction. Participants believed that the interactive website provided them with a valuable opportunity to improve their knowledge of how to strengthen their social networks in a broad space for efficient communication. A novice English teacher commented that ‘though face-to-face teaching models are still dominant, using Web 2.0 technologies are easier for younger generations to get authentic English resources’. Another participant believed that the project website provided them with an efficient way to engage with others, maintain connections, and obtain the latest information. In addition, she further explained her personal reasons why she would like to be part of a broader community:

*I am a mum with two kids, and I usually consider how to develop myself in the limited spare time after work. This website allows me to link my own Facebook account to share information with other inexperienced peers, and questions were answered quickly. Being a mum and a teacher, I used it not only to interact with peers but also to engage with other parents who are in a similar context as me. They might come from different language backgrounds, and I strongly believe this experience could help me to develop my teaching skills in a multicultural environment.* (P5 interviewee)

“The project website that links with my personal social media account allowed me to obtain job vacancy information on external websites,” reflected one female teacher who had just started her career. She responded that the social media tools she used in daily life might not be specific to her career information, as most of them focus on marketing and business; thus, people who had been involved in sharing information on the project website could be their potential colleagues or employers in the near future. Furthermore, she commented that her intercultural awareness has been enhanced by her acquisition of resources shared by the teachers who were involved in the community.

Other appraisals of adoption the project website in language classrooms were grouped as “Mobile-friendly” (*n* = 18), ‘Compatible with systems’ (*n* = 15), and “Easy to use” (*n* = 14). A male interviewee (P2) reflected that the technology infrastructures used in these schools were more modern and advanced than those used in other public schools due to foreign investments. He responded that novice English teachers used the same teaching materials as required by their overseas partners. In this case, he believed that the adoption of the project website for use in the process of interaction would be easy for him and that the website was compatible with mobile devices as well. He further commented that the operating system would be an influential factor for him in deciding which social media tools he would like to use, as he only had access to an IOS operating system.

### Concerns regarding the adoption of social media technologies

Participants’ concerns were primarily categorised as “Confidentiality” (*n* = 57), “Lack of digital literacy skills” (*n* = 40), and “Interface design” (*n* = 37). A large number of comments from the open-ended questions in the questionnaire reflected concerns regarding source and data confidentiality. An example participant’s response is shown below:

*I have realised the importance of using Web 2.0 technologies in teaching and learning, but I do not feel comfortable using them in language classrooms. This might be due to school data security. If I post students’ assignments or their news on the project website, I am not sure if they would be happy to have them shared. The information could be related to school policies, teaching materials used by colleagues and recordings. Even this is only for research purposes, but I still worry about it.* (Open-ended question response)

Participants indicated that a lack of e-learning design skills and the unattractive interface design of the website might influence their decisions regarding the adoption of Web 2.0 technologies in language classrooms. A female interviewee shared her negative experiences with the researcher in the interview as follows:

*I completely understand that it is essential for language teachers to develop their awareness of using Web 2.0 technologies in classrooms. However, most of my colleagues and I were not confident in using those tools efficiently in language teaching. For example, I do not know how to embed the online resources successfully in my current teaching materials. Teachers were pushed to teach everything that was based on language textbooks, and students had to finish assignments. I am not sure if these would efficiently help them in a 45-minute English class, as students’ performance was evaluated by examinations. Additionally, the website design is not very attractive. The interface is a bit boring. If the design were more engaging, there would be more people involved*. (P6 interviewee)

### Professional development for novice teachers

This category was classified as the third theme. Although there were not as many responses as for the first two categories, there were still two subgroups that could be identified to represent participants’ perceptions: a lack of internal support (*n* = 20) and a lack of external support (*n* = 12) for novice teachers’ development. Most interviewees indicated that after their former employment, they were not required to finish further professional development programs, unlike teachers in the United Kingdom, Australia, and the United States. An interviewee expressed that she might not have known any innovative teaching methods and strategies if she were not required to attend professional development programs. She believed that this was normal for novice teachers at public schools in the Czech Republic. She further commented that the reasons for this might include a lack of financial support from schools, local organisations, and communities. A male interviewee expressed his view that both internal and external support is important for novice teachers if they intend to attend professional development programs. He gave an example in his school:

The school principal encouraged us to attend professional programs, as I am in an international teaching environment. I was provided with a little bit of funding to support me in joining a range of Language Teachers’ Associations. I could have free access to the online resources and communicate with peers in forums. However, when I wanted to use innovative (new) teaching strategies in my classroom, it was difficult to gain support from parents and local communities. We need to improve parents’ understanding, and sometimes, it is not an easy task for me.

Some participants believed that it would be helpful if they received support to attend professional development programs. This was evidenced from participants’ responses in the open-ended questions, as in the following example:

I am busy with my job and looking after my family. I am considering if I could be financially supported by the school to attend the online programs. This is important for language teachers to develop and obtain information from English-speaking countries.

## Discussion

Based on the results from both the quantitative and qualitative stages, we found that the novice EFL teachers in this study had a strong understanding of Web 2.0 technologies and were aware that the adoption of Web 2.0 technologies is important for both teachers and learners to communicate and interact in a board community. This result is in contrast with previous research, which claims that teachers in less developed areas are “pre-digital” and not informed (Selwyn et al., 2018). These inconsistent results might be due to the participants’ learning and teaching contexts, both historical and linguistic. Historically, Czech teachers have been trained in a post-soviet educational system, which provided teaching and learning resources in a monolingual way. The majority of migrants were from Slavic countries. Hence, compared with teachers from the United Kingdom and Australia, the teachers in the study have not been provided many opportunities to be involved in a multicultural teaching environment. Generally, the project website provided an opportunity for them to seek information and interact with peers, which could enhance their teaching and learning skills in their professional jobs. This finding is consistent with those of other studies (Yost and Fan, 2014; Authors, 2015; Carpenter et al., 2016; Lau, 2018; Godhe et al., 2020; Lu, 2022). Web 2.0 technologies have revolutionised communication and the dissemination of information (Holt, 2011). People merely need to click navigation bars to obtain a large amount of information, communicate and interact in a timely manner, and be instantly engaged in the broad community. In addition to its friendly user interface and instant communication, the interviewees noted that being engaged in the broader community, as the project website offers, may also support language teachers’ further involvement in the multicultural community. This result accords with research studies that focused on creating an intercultural community by using social media (Veronis et al., 2018).

Support for being involved in an online community indicates that participants have a strong willingness to communicate and develop their skills in this informal way due to the low cost and high efficiency. This result is similar to that of studies that highlighted the advantages of the development of online informal professional communities using Web 2.0 technologies (Marklund, 2015; Lantz-Andersson et al., 2018). In the study, participants who had a higher self-reported ICT proficiency level felt more confident in using social media in language classrooms, and vice versa. As a consequence, their ICT proficiency influences their motivation and willingness in relation to the adoption of social media tools for teaching and learning. This finding supports previous studies undertaken in Asia, which indicated that teachers’ motivation and intention are essential metrics to measure whether the usage of social media in language teaching will be successful (Mei et al., 2018).

The participants mostly expressed concerns regarding confidentiality. In this study, the context involved school policies and information from students and colleagues. As recognised in previous research (Lai et al., 2011; Yost and Fan, 2014; Li et al., 2019), the participants were also concerned about personal data security. School teachers need to be responsible for managing students’ profiles and reporting their learning outcomes to parents. In this process, parents might be interested in sharing their children’s information with others. In such a case, the use of social media tools was conditional and determined in accordance with the extent of security and privacy that might be afforded. Except for this reason, most novice English teachers showed a strong willingness to spend their spare time interacting with parents and more experienced teachers in the multicultural online community, which does not accord with the results of previous studies (Rosenberg et al., 2016) that have argued that ‘work overload’ and ‘limited spare time’ influence teachers’ engagement in online communities as well as their adoption of Web 2.0 technologies in the Czech context (Kutálková, 2017).

### Limitations

This study has several limitations. First, it is an explorative study, and the majority of participants were from the two largest regions in the Czech Republic: the Prague and South Moravian regions have larger populations and potentially a better economic status than West Bohemia and rural regions. Future studies could focus on in investigating teachers’ digital habitats in less developed regions, which might generate more interesting results. Second, we did not explore more experienced EFL teachers’ digital habitats due to our limited research budget. A comparison of novice and experienced teachers’ digital habitat might assist researchers in developing a better understanding of the disparity of their behaviour and preferences in regard to using social media web 2.0 tools in classroom teaching. As a consequence, this may help decision-makers initiate English cubiculum reform in the Czech context.

### Implications

The findings from this study have both political and practical implications. Teachers’ having conservative attitudes is not beneficial for the innovative curriculum form. In this study, a number of significant policy issues and practise implications were found relating to English learning and teaching in K–12 education in the Czech Republic. Two prominent implications were discovered: the EFL curriculum in the Czech educational system and raising awareness regarding the use of social media in language teaching in multicultural schools. Currently, English is a compulsory subject taught as a foreign language in K–12 education. According to the Ministry of Education Youth and Sports (2007), the requirements for basic English language education are based on the Common European Framework and include achieving a English satisfactory level (A1 or A2). Additionally, the language curriculum used in individual schools should be developed based on the National Educational Programs. In this case, enacting any innovative reforms regarding the development of EFL curriculum, teaching methods, and teaching materials is difficult due to restrictive school management. EFL teachers must follow the traditional ways of teaching students face to face, and students are primarily evaluated *via* written assignments and final examinations, which may lead to the devaluation of the adoption of social media tools in language classrooms, as it does not yield direct benefits to schools, teachers, or students. Moreover, many teachers have few opportunities to interact and communicate efficiently with parents in multicultural families due to the lack of digital technologies. Beran et al. (2007) claimed that financial support is essential when technology equipment is needed in classrooms.

In teaching practise and daily management, supervisors’ management and teachers’ development programs should be developed at the school, state, and national levels. At present, fully employed novice EFL teachers are not provided with mentors, which results in a lack of supervision by the schools. In this case, inexperienced teachers may easily feel vulnerable if they cannot obtain sufficient support at work. As a consequence, teachers’ stress and other negative emotions can influence students’ cognitive development, such as professional development of the skills essential to employ social media tools in EFL classrooms (Hayes, 2003). According to Bakkenes et al. (2010), innovations in school have often failed because teachers’ learning and development have not been given sufficient attention. In this regard, teachers need to be supervised by more experienced mentors on a regular basis to develop language teaching skills both online and offline. Herout (2017) argues that social media tools should be embedded in formal education and daily teaching because of their popularity among the digital generations. Additionally, in a global nexus, using social media tools in language classrooms is beneficial for the development of students’ intercultural awareness and multilingualism as well as their multicultural identity. It is urgent for decision-makers to consider these potential factors in the new round of language curriculum development and reform; otherwise, EFL teaching methods, teachers’ digital literacy skills, and language evaluation could be left far behind those in developed countries in Europe.

## Conclusion

This paper examines novice English teachers’ perceptions of how one type of social media technology, Web 2.0 technology, can be used to facilitate collaboration and communication in an EFL teacher community in the Czech Republic. Generally, teacher held a positive attitude towards the adoption of social media tools in interaction, communication, and professional development. Concerns remained regarding data confidentiality and e-learning design skills. Notably, novice EFL teachers’ ICT proficiency level is a factor influencing the participants’ understanding, awareness, and concerns regarding their digital habitats. The higher the level of their ICT proficiency, the fewer concerns teachers have. However, the reasons why participants with higher levels of ICT proficiency show more interest in training or further development programs using Web 2.0 technologies remain unclear in the current study. We suggest that future research studies be developed to investigate factors that correlate with teachers’ decisions to use social media tools in English language teaching in the Czech Republic.

## Data availability statement

The raw data supporting the conclusions of this article will be made available by the authors, without undue reservation.

## Ethics statement

The studies involving human participants were reviewed and approved by the Ethics Committee at Masaryk University (MUNI). The ethics committee waived the requirement of written informed consent for participation.

## Author contributions

JL conceptualized, collected, and analyzed the data and drafted the paper. FH completed the quantitative data analyses and revised the survey and the draft. TJ provided the guidance and revised the paper. All authors contributed to the article and approved the submitted version.

## Conflict of interest

The authors declare that the research was conducted in the absence of any commercial or financial relationships that could be construed as a potential conflict of interest.

## Publisher’s note

All claims expressed in this article are solely those of the authors and do not necessarily represent those of their affiliated organizations, or those of the publisher, the editors and the reviewers. Any product that may be evaluated in this article, or claim that may be made by its manufacturer, is not guaranteed or endorsed by the publisher.

## Footnotes

^1^In the Czech Republic, students have to complete 9 years of compulsory education, usually from the ages of 6–15 and mostly at a basic school (*základní škola*). Pupils can leave the basic school at the end of their fifth year for an 8-year *gymnázium* or at the end of their seventh year for a 6-year *gymnázium* after passing the entrance examination set by the school. There are three main types of secondary schools in the Czech Republic: general secondary school (*gymnázium*), secondary technical school (*střední odborná škola—SOŠ*), and secondary vocational school (*střední odborné učiliště—SOU*; https://www.mzv.cz/dublin/en/about_the_czech_republic/education_in_the_czech_republic/).
